# Human umbilical cord mesenchymal stem cell promotes angiogenesis via integrin β1/ERK1/2/HIF-1α/VEGF-A signaling pathway for off-the-shelf breast tissue engineering

**DOI:** 10.1186/s13287-022-02770-x

**Published:** 2022-03-07

**Authors:** Mian Wu, Lifeng Chen, Yuhan Qi, Hai Ci, Shan Mou, Jie Yang, Qiaoyu Yuan, Weiqi Yao, Zhenxing Wang, Jiaming Sun

**Affiliations:** 1 Department of Plastic Surgery, Union Hospital, Tongji Medical College, Huazhong University of Science and Technology, Wuhan, 430022 People’s Republic of China; 2 Department of Thyroid and Breast Surgery, The Central Hospital of Wuhan, Tongji Medical College, Huazhong University of Science and Technology, Wuhan, 430014 People’s Republic of China; 3 Wuhan Clinical Research Center for Superficial Organ Reconstruction, Wuhan, 430022 People’s Republic of China; 4 Wuhan Optics Valley Zhongyuan Concord Cell Gene Technology Co., Ltd, Wuhan, People’s Republic of China; 5 National Industrial Base for Stem Cell Engineering Products, Tianjin, People’s Republic of China; 6 Department of Hematology, Union Hospital, Tongji Medical College, Huazhong University of Science and Technology, Wuhan, 430014 People’s Republic of China

**Keywords:** Tissue engineering, Mesenchymal stem cells, Umbilical cord, Angiogenesis

## Abstract

**Background:**

Mesenchymal stem cells (MSC)-based tissue engineered breast represent the visible future for breast reconstruction after mastectomy. However, autologous MSCs might not be appropriate for the large graft construction due to cell senescence during excessive cell expansion, thus hindering its further off-the-shelf application. The human umbilical cord mesenchymal stem cells (hUCMSCs) have been found to induce low immune response and can be easily stored, making them ideal for off-the-shelf tissue engineering application. Here, we explored the feasibility of using umbilical cord mesenchymal stem cells as tissue-engineered breast seed cells.

**Methods:**

The allogenic hUCMSCs were injected into transplanted fat tissue with or without breast scaffolds as an alternative for breast tissue engineering in vivo, and its potential mechanism of angiogenesis in vitro was explored.

**Results:**

Transplantation of hUCMSCs promoted proliferation, migration, and angiogenesis of human umbilical vein endothelial cells (HUVECs) through paracrine mechanism by activating the integrin β1/ERK1/2/HIF-1α/VEGF-A signaling pathway. Histological examination of grafted fat revealed that the group which received hUCMSCs transplantation had more fat tissue [(93.60 ± 2.40) %] and fewer MAC2^+^CD206^−^ M1 macrophages [(0.50 ± 0.47) cells/field] compared to the control group [fat tissue (45.42 ± 5.96) and macrophage cells/field (5.00 ± 2.23)]. Moreover, the hUCMSCs- labeled with a tracing dye differentiated into adipocytes and vascular endothelial cells in the adipose tissue. When applied to the tissue-engineered breast with a scaffold, the group treated with hUCMSCs had more adipose tissues and CD31^+^ cells than the control group.

**Conclusions:**

These results demonstrate that allogeneic hUCMSCs promote the regeneration of adipose tissue and can be used to construct a tissue engineered breast.

**Supplementary Information:**

The online version contains supplementary material available at 10.1186/s13287-022-02770-x.

## Introduction

Breast cancer is the most prevalent cancer among women worldwide. Breast reconstruction restores body image and improves the quality-of-life post-mastectomy [1]. In traditional surgical strategies, a breast is reconstructed using a flap or an implant. Traditional surgical strategies result in donor site morbidity and capsule contraction. These drawbacks can be resolved by restoration of the breast shape through tissue engineering. Formation of a stable soft tissue on the recipient site requires sufficient vascularization and adipose tissue regeneration [2, 3], and this is a major challenge in breast tissue engineering. Stem cells act as seed cells in tissue engineering whereby they can regenerate expected tissue via direct differentiation into proper cell type and paracrine regulation [4, 5].

In recent years, adipose-derived stem cells (ADSCs) have been successfully applied in breast tissue construction [6]; however, the use of ADSCs faces several potential limitations. Generally, ADSCs are obtained through liposuction, which causes new trauma. The number of cells and their potential to proliferate and differentiate are related to the extraction site, the age of the donor, and the state of the entire body [7]. Human umbilical cord mesenchymal stem cells (hUCMSCs) were isolated in 2003 [8]. Since then, researchers have demonstrated several benefits of these cells. For instance, hUCMSCs derived from discarded tissue of newborns are rich in sources. hUCMSCs also exhibit high proliferative capability, low immunogenicity, and multilineage differentiation potential [9–11]. However, whether hUCMSCs can be applied in breast tissue engineering has not been fully clarified.

Rapid and efficient vascularization is an important factor that determines the success of breast tissue engineering given the hypoxic conditions in the adipose tissue. HUCMSCs have been shown to promote vascularization in ischemic diseases, including acute mesenteric ischemia [12], ischemic stroke [13], flap ischemia [14], lower limb ischemia [15], etc. Recent research has strongly recognized the potential role of angiogenesis via paracrine secretion. For instance, the conditioned medium (CM) or their extractive of hUCMSCs could induct vascularization in ovarian cortical tissue [16], damaged cardiomyocytes [17], and spinal cords [18]. VEGF-A is among the most crucial angiogenic factors because it regulates the formation of new blood vessels [19]. Studies indicate that hUCMSCs can induce high expression of VEGF-A; however, the actual mechanism is elusive. Integrins, a family of cell adhesion receptors, play significant roles in cell–cell communication and modulation of various signaling pathways, such as angiogenesis [20]. In this study, we investigated whether hUCMSCs promote angiogenesis by activating integrin via a paracrine mechanism.

A well-engineered breast provides stable soft tissue with favorable adipogenesis. The differentiation and proliferation of stem cells is influenced by the microenvironment, also called the “stem cell niche” [21]. Most recently, a study revealed that hUCMSCs could differentiate into odontoblast-like cell via human dentin matrix [22]. The inserted adipose tissue also underwent neoadipogenesis, which was potentially driven by the stem cell and native adipose extracellular matrix. Elsewhere, Yao, Y confirmed that the adipocyte-free liposuction could induce host cell-mediated adipogenesis [23]. Based on these findings, we aimed to explore whether hUCMSCs can be induced to differentiate into adipocytes when transplanted on adipose tissue.

Previously, we explored the actual structure of the 3D printing scaffold applied in breast tissue engineering [24]. This study explores whether hUCMSCs can serve as seed cells for breast tissue engineering and elucidate the underlying mechanism. The effects of hUCMSCs on the physiological function of HUVECs and the associated signaling pathways were explored. HUCMSCs were then co-transplanted with human fat graft with or without breast scaffold into nude mice for three months. This allowed us to explore the effects and mechanisms of hUCMSCs transplantation on tissue-engineered breast construction.

## Materials and methods

### Adipose tissue harvesting

Liposuction aspirates were obtained from healthy female donors undergoing liposuction of the thighs at the Department of Plastic Surgery, Wuhan Union Hospital (Wuhan, China). The protocol was approved by the Ethics Committee of Huazhong University of Science and Technology (Wuhan, China). Aspirated fat tissues were collected immediately after liposuction and then washed by normal saline for more than five times until the water layer was colorless. After centrifuged at 3000 rpm for 5 min, the middle fat layer was collected for further use.

### Immunophenotyping

hUCMSCs isolated from consenting full-term caesarean section patients were obtained from Wuhan Optics Valley VCANBIO Cell Gene Technology Co., Ltd. (Tianjin, China). Cells were cultured at 37 °C in 5% CO_2_ 95% air-humidified incubator, harvested using 0.05% trypsin, centrifuged and resuspended in hUCMSCs complete medium (Cyagen Biosciences, USA). To identify the specific cellular surface markers CD29, CD44, CD45, CD90 and CD105, immunofluorescence staining was performed on hUCMSCs in passage 6. The primary antibodies were rabbit anti-human CD29, CD45 monoclonal antibodies, mouse anti-human CD44, CD90 and CD105 monoclonal antibodies (1:100; Abcam, Cambridge, UK). And the secondary antibodies were rabbit anti-mouse immunoglobulin G conjugated with fluorescein isothiocyanate (Abcam). 4′,6-Diamidino-2-phenylindole (Abcam) was used for nuclear staining.

### Cell viability assay

A total of 1 × 10^4^ hUCMSCs per well were seeded onto a 96-well plate (five wells per group). At 1, 3, 5, 7, 9, and 11 days, cell viability was detected using a Cell Counting Kit-8 (Dojindo Molecular Technologies, Kumamoto, Japan), according to the manufacturer’s protocol.

### The experiment of influences of hUCMSCs on HUVECs in vitro

#### Transwell cell migration assay

Human umbilical vein endothelial cells (HUVECs) were acquired from the American Type Culture Collection (Rockville, Md.) and incubated in culture medium at 37 °C in a humidified atmosphere with 5% carbon dioxide. A Transwell system was used to estimate the migration ability of HUVECs in vitro. The bottom of the culture inserts (8-mm pores) in 24-well tissue culture plates (Transwell; Corning) was coated with serum-free medium at 37℃ for 1 h. Eight chambers were randomly divided into two groups (*n* = 4 per group): (1) Control group and (2) hUCMSC group. hUCMSCs were harvested by trypsinization and then washed by serum-free medium. In hUCMSC group, hUCMSCs were seeded in the lower chamber with 3 × 10^4^ cells/well separated in 800 μl serum-free medium. No cell was seeded in the lower chamber in the Control group. Waited overnight for hUCMSCs adherence. Then, HUVECs were added into all the upper chambers with 3 × 10^4^ cells/well suspended in 200 μl serum-free medium. After 24 h of incubation at 37℃ with 5% CO_2_, the upper chambers were taken out, washed gently with sterile PBS and fixed in 4% paraformaldehyde for more than half an hour. Then, a cotton swab was used to gently wipe the cells in the chamber that did not migrate to the basal side of the membranes, and crystal violet solution was used for staining. The cell morphology in each upper chamber was observed under an inverted microscope. The numbers of cells in each well migrating to the basal side of the membrane were quantified by counting 3 random symmetrical visual fields. Double blind method was used in the choice of field of vision and data statistics.

#### Transwell cell proliferation assay

HUVECs suspended in serum-free medium were seeded with 1 × 10^4^ cells/well in the lower chamber of a 24-well tissue culture plates and waited overnight for hUCMSCs adherence. Twenty-four chambers were randomly divided into two groups (*n* = 12 per group): (1) Control group and (2) hUCMSC group. hUCMSCs in serum-free medium with 3 × 10^4^ cells/well was seeded in each upper chamber in the hUCMSC group, and only serum-free medium was added in the Control group. For the next three days at the same time point, cells of HUVECs were counted after trypsin digestion in the lower layers of 4 random chambers in each group. Double blind method was adopted in the data statistics of this experiment.

#### Tube formation assay

Once a 10-cm culture plate of hUCMSCs in passage 6 reached 60% confluence, discarded the original culture medium and added 7 ml fresh hUCMSCs complete medium. The culture medium was collected 3 days later. The cell fragments were filtered out with a filter, and then, the medium was centrifuged with 4000r for 20 min. The bottom of the culture medium was collected 1 ml, named as hUCMSC-cm. Matrigel (BD Biosciences, San Jose, Calif.) was added to a 96-well culture plate. Twelve wells were randomly divided into two groups (*n* = 6 per group): (1) Control group and (2) hUCMSC group. HUVECs in the Control group were seeded with 2 × 10^4^ cells/well in fresh hUCMSCs complete medium, while HUVECs in the hUCMSC group were seeded with 2 × 10^4^ cells/well in hUCMSC-cm. The plate was incubated at 37 °C in 5% CO_2_ 95% air-humidified incubator for 6 h. Tube formation was assessed under a phase-contrast microscope. Six fields were randomly selected from 12 fields of each group to make statistics on the number of tubes, and the total length of tubes was measured and counted by Image J software. Double blind method was used in the choice of field of vision and data statistics.

#### Binding assays

Once a 10-cm culture plate of hUCMSCs in passage 6 reached 80% confluence, discarded the original culture medium and added 7 ml fresh hUCMSC serum free (SF) medium. The medium was collected 24 h later and named as hUCMSC conditioned medium (CM). A million HUVECs were plated in each 10-cm Petri dish and serum starved for 24 h before treatment with ATN-161 (50 μM) or 2-MeOE_2_ (50 μM) for 30 min, then changed the medium to hUCMSC-conditioned medium for 24 h, washed gently with sterile PBS, then harvested these HUVECs, and named as ATN-161 group or 2-MeOE_2_ group. For SF group, HUVECs serum starved for 2 days before harvest. For CM or RPMI group, HUVECs starved for 24 h before treatment with hUCMSC-conditioned medium or RPMI medium for 24 h (Fig. 2A).

#### Experiment of signaling pathway

Western blot analyses were carried out using primary antibodies against HIF-1α (1:1000, #ab2185, Abcam), VEGF-A (1:1000, #19003-1-AP, PTG), ERK1/2 (1:1000, #4695, CST), phospho-ERK1/2 (1:1000, #4370, CST), Akt (1:2000, #9272, CST), phospho-Akt (1:1000, #9271, CST), and β-actin (1:8000, #KM9001, Tianjin Three Arrows). Secondary antibodies were combined with corresponding primary antibody. The immune complexes were visualized by the enhanced chemiluminescence detection system according to the manufacturer’s protocol. Double blind method was used in measuring integrated optical density (IOD) by Image J software. To test the migration abilities of HUVECs in different groups, the method was almost the same as mentioned above other than all the lower chambers were added 800 μl hUCMSC-conditioned medium. The same method mentioned above was used to explore the tube formation abilities of differently treated HUVECs.

### Experiment of hUCMSCs transplantation into fat graft without breast scaffold

#### Animal model

Animal studies in this article were performed in accordance with the guidelines of the Ethics Committee of Huazhong University of Science and Technology. Female 6-week BALB/c-nu nude mice were obtained from Beijing Vital River Laboratory Animal Technology Corp., Ltd. (Beijing, China). To investigate the effects of hUCMSCs on the volume retention of fat grafts in vivo, mice were randomly divided into four groups (*n* = 4 per group): (1) Control group; (2) Low hUCMSC group; (3) Medium hUCMSC group; and (4) High hUCMSC group. After anesthetized by inhaling 3% isoflurane, the recipient mice in the Control group were injected subcutaneously with 0.5 ml human fat on each side of the back using an 18-gauge needle. In three hUCMSC groups, mice were injected subcutaneously using the same method on each side of the back with 0.5 ml of human fat before multipoint injection of 50 µl of sterile saline containing 0.5 × 10^6^ hUCMSCs, 1.0 × 10^6^ hUCMSCs or 2.0 × 10^6^ hUCMSCs, respectively. After 12 weeks of the implantation, all fat grafts were harvested, and their volumes were measured by liquid overflow method. The harvested sample volume to the initial volume (0.5 ml) was used to normalize the retention ratio. Each sample was fixed in 4 wt% paraformaldehyde (PFA) in 0.1 m phosphate-buffered solution overnight and embedded in paraffin. Each sample was subjected to at least six tissue slices.

#### Histological evaluation

Two sections were randomly selected from those slices in each sample. Tissue sections were stained with hematoxylin–eosin using standard procedures and examined under a light microscope (Nikon E600; Nikon Corp., Tokyo, Japan).

Six random fields at 4 × magnification in each group were selected by a single blinded observer. Histology scores were generated by another two independent and blinded observers and averaged for each group. The scoring method was based on a previously published scale [25], which assessed the presence of intact and nucleated fat cells; presence of cysts and vacuoles; inflammation, as evidenced by infiltration of lymphocytes and macrophages; and presence of fibrosis and other components of the connective tissue (i.e., collagen and elastic fibrils). Each score was evaluated based on the following scale: 0 = absence, 1 = minimal presence, 2 = minimal to moderate presence, 3 = moderate presence, 4 = moderate to extensive presence, and 5 = extensive presence. The ratio of fibrosis, necrosis and fat area to total tissue area were evaluated using ImageJ (Version 1.46 software, NIH) by another single blinded observer.

Six random fields at 10 × magnification in each group were selected by a single blinded observer. Twenty-five random adipocytes were selected in each field, and the diameters of these cells were measured by a second single blinded observer using ImageJ.

#### Immunofluorescence assessment

Four sections were randomly selected from those slices in each sample, and the section was double-stained with the following primary antibodies: rabbit anti-human Perilipin (Cell Signaling Technology) and mouse anti-human CD31 (Abcam), mouse anti-human MAC2 (Abcam) and rabbit monoclonal CD206 (Abcam). Nuclei were stained with DAPI.

For Perilipin and CD31 immunofluorescence double staining, eight fields under the fluorescence microscopy at 10 × magnification in each group were randomly selected by a single blinded observer. Positively stained vessels were measured by a second blinded observer using ImageJ (Version 1.46 software, NIH). Threshold values were established from subtracting background pixel count using the control. Relative mean pixel counts from groups of slides were averaged, and the standard deviation was calculated.

For MAC2 and CD206 immunofluorescence double staining, another eight fields under the fluorescence microscopy at 20 × magnification in each group were randomly selected by a single blinded observer. The numbers of MAC2^+^ CD206^−^ cells and MAC2^+^CD206^+^ cells were calculated independently by a second blinded observer.

#### CM-Dil cell tracing experiment

hUCMSCs were prelabeled by incubation with the carbocyanine fluorescent dye CM-Dil (Molecular Probes, Carlsbad, CA) for 30 min. Four nude mice were injected subcutaneously on each side of the back with 0.5 ml human fat and 50 µl of sterile saline containing 0.5 × 10^6^ CM-Dil-labeled hUCMSCs. After 12 weeks of the implantation, the samples were harvested and embedded in OCT compound (Miles, Elkhart, IN), snap-frozen in liquid nitrogen and then cut into 5-mm-thick sections. Viable hUCMSCs were identified by localizing CM-Dil. Sections were stained with Perilipin or CD31 primary antibodies to detect capillary endothelial cells or adipocytes. Nuclei were stained with DAPI.

### Experiment of hUCMSCs transplantation into fat graft with breast scaffold

#### Animal model

Experimental mice were randomly divided into two groups (*n* = 4 per group): (1) Control group and (2) hUCMSC group. After anesthetized by inhaling 3% isoflurane, the recipient mice in the Control group were implanted subcutaneously with the scaffold filled with 0.2 ml human fat on each side of the back. In the hUCMSC groups, mice were implanted subcutaneously using the same method on each side of the back with scaffold filled with 0.2 ml of human fat and 20 µl of sterile saline containing 0.2 × 10^6^ hUCMSCs. The material and architecture of scaffold in the present study refer to our previous research [24]. The scaffold is built with N5S4 architecture which mimic the lattice microstructure of diamond are soft and resilient enough for breast tissue engineering, which has showed considerable advantages in animal study. After 4 or 12 weeks of the implantation, all compounds were harvested. The weights were determined by a balance and their volumes were measured by liquid overflow method. Each sample was fixed in 4 wt% PFA in 0.1 M phosphate-buffered solution overnight and embedded in paraffin. Each sample was subjected to at least six tissue slices.

#### Histological evaluation

Two sections were randomly selected from those slices in each sample. Tissue sections were stained with hematoxylin–eosin using standard procedures and examined under a light microscope.

Four random fields at 4 × magnification in each group were selected by a second single blinded observer. The ratio of fibrosis, necrosis and fat area to total tissue area were evaluated using ImageJ (Version 1.46 software, NIH) by another single blinded observer. The total tissue area was calculated by the total area minus the total scaffold area.

Fibrotic capsules could be identified as a dense layer of collagen fibers aligned parallel to the implant surface with a variable presence of fibroblasts and inflammatory cells in HE staining sections [26]. Three random fields at 4 × magnification in each group were imported in ImageJ and measurement lines, approximately 50 μm apart, were defined on the fibrotic capsule. Average thickness of the fibrotic capsule was derived from the average of such measurement lines.

#### Immunofluorescence assessment

Four sections were randomly selected from those slices in each sample, and the section was stained with the following primary antibodies: mouse anti-human CD31, mouse anti-human CD68. Nuclei were stained with DAPI.

For CD31 immunofluorescence staining, six fields under the fluorescence microscopy at 10 × magnification in each group were randomly selected by a single blinded observer. Positively stained vessels were measured by a second blinded observer using ImageJ. Threshold values were established from subtracting background pixel count using the control. Relative mean pixel counts from groups of slides were averaged and the standard deviation was calculated.

For CD68 immunofluorescence staining, another six fields under the fluorescence microscopy at 20 × magnification in each group were randomly selected by a single blinded observer. The numbers of CD68^+^ cells were calculated independently by a second blinded observer.

### Statistical analysis

All statistical data were expressed as the mean ± SD. The data were analyzed by t test or ANOVA to determine the statistical significance. *p* < 0.05 was defined to be significant. All statistical analyses were performed using the GraphPad Prism (GraphPad Software, Inc., San Diego, Calif.).

## Results

### Effects of hUCMSCs on HUVECs in vitro

**Fig. 1:**
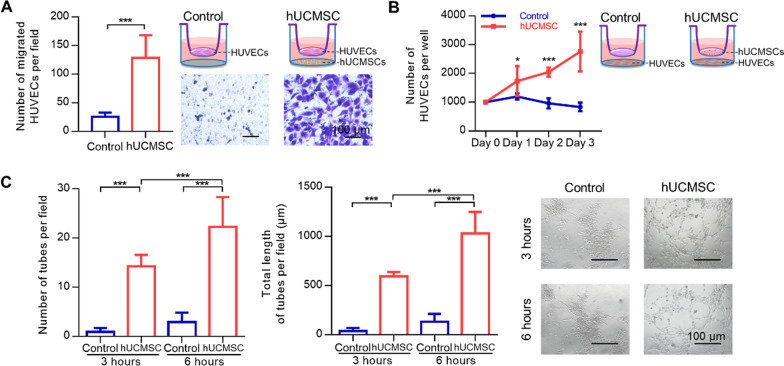
HUCMSC-CM promoted HUVECs migration, proliferation, and tubule formation in vitro. The conditional medium of HUCMSCs could promote the migration (**A**), proliferation (**B**) and tubule formation (**C**) of HUVECs. Scale bars = 100 μm

The hUCMSCs were positive for CD29, CD44, CD90, CD105, but negative for CD45 (Additional file 1: Fig. S1A). High passage (P8) hUCMSCs exhibited efficient proliferative capacity (Additional file 1: Fig. S1B). Firstly, the effects of hUCMSCs on endothelial cell migration and angiogenesis were test using transwell assays and tube formation assay. In Transwell systems, hUCMSCs potentially promoted the migration of HUVECs (Fig. 1A). In the presence of hUCMSCs, HUVECs in the serum-free medium changed from small and round into large polygon morphologically. hUCMSCs could also promote the proliferation of HUVECs in the serum-free medium (Fig. 1B). After culturing hUCMSCs, the medium could increase the number and total length of HUVECs-derived tubes, which were more than seven times larger (Fig. 1C). Using the tube formation assay, similar morphological changes of HUVECs were reported in the hUCMSC group.

### hUCMSCs promote angiogenesis by activating the integrin β1/ERK1/2/HIF-1α/VEGF-A signaling pathway

**Fig. 2:**
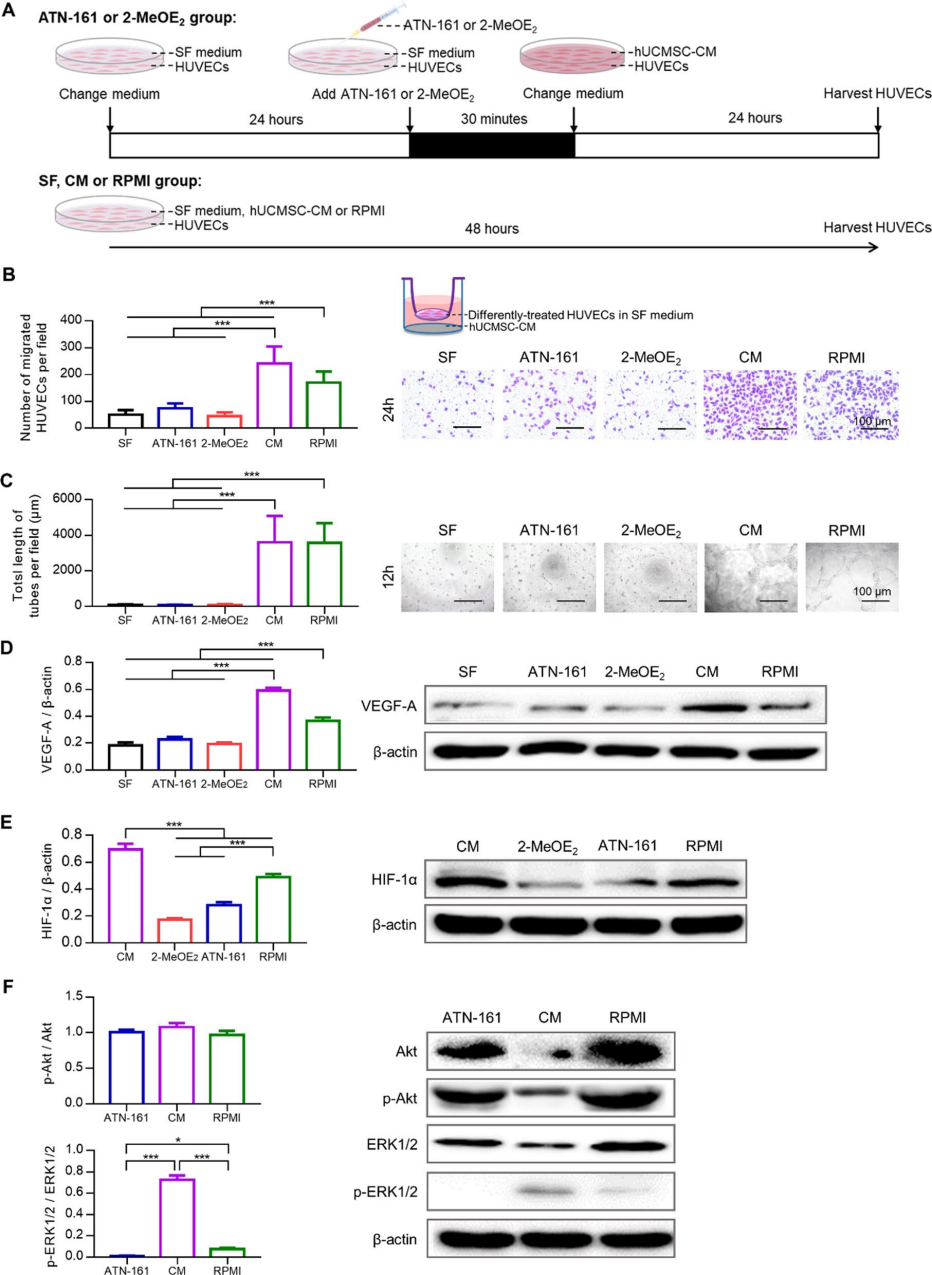
HUCMSC-CM promoted migration and tubule formation via integrin β1/ERK1/2/HIF-1α signaling pathway in HUVECs. **A** ATN-161 or 2-MeOE_2_ was used to pre-treat HUVECs to inhibit integrin β1 or HIF-1α, respectively. **B**, **C** HUVECs migration and tubule formation were observed after different treatments. ATN-161 or 2-MeOE_2_ significantly inhibited hUCMSC-CM-induced migration and tubule formation in HUVECs (***P < 0.001). **D** Western blot analysis showed hUCMSC-CM-induced VEGF-A expression was significantly suppressed following ATN-161 or 2-MeOE_2_ treatment in HUVECs (***P < 0.001). **E** Western blot analysis showed hUCMSC-CM-induced HIF-1α expression was markedly decreased following integrin β1 inhibition in HUVECs (***P < 0.001). **F** hUCMSC-CM significant promoted ERK1/2 phosphorylation in HUVECs and could be suppressed by inhibition of integrin β1. In contrast, hUCMSC-CM did not affect Akt phosphorylation in HUVECs (***P < 0.001)

VEGF-A plays a vital function in both vasculogenesis (de novo vasculature formation) and angiogenesis (vessel formation sprouting from existing vasculature) [27]. The expression of VEGF-A is upregulated by HIF-1α [28]. Besides, it has been reported that integrinβ1 was closely related to VEGF-A and could contribute to VEGF-A-induced angiogenesis [29]. Thus, we tested the connection between integrin β1, HIF-1α and VEGF-A. ATN-161 is a peptide that binds to integrin β1 [28], whereas 2-MeOE_2_ is an inhibitor of HIF-1α [30]. The migration and tube formation abilities of HUVECs were weakened following treatment with ATN-161 and 2-MeOE_2_, which was comparable to treatment in serum-free medium (Fig. 2B, C). Compared to when the RPMI medium was used, the expressions of HIF-1α and VEGF-A in HUVECs pretreated with ATN-161 or 2-MeOE_2_ were significantly lower. The levels of HIF-1α and VEGF-A were significantly higher when HUVECs were cultured in hUCMSCs conditioned medium (Fig. 2D, E). Additionally, we found no significant difference in VEGF-A expression between the SF, ATN-161, and 2-MeOE_2_ groups (Fig. 2D). p-Akt/Akt exhibited no significant difference between different groups, whereas the ratio of p-ERK1/2 to ERK1/2 in the CM group was significantly higher compared to the other groups (Fig. 2F). However, following treatment with ATN-161, the ratio was significantly lower compared to when we used the RPMI medium.

### hUCMSC-assisted Lipotransfer in vivo

**Fig. 3:**
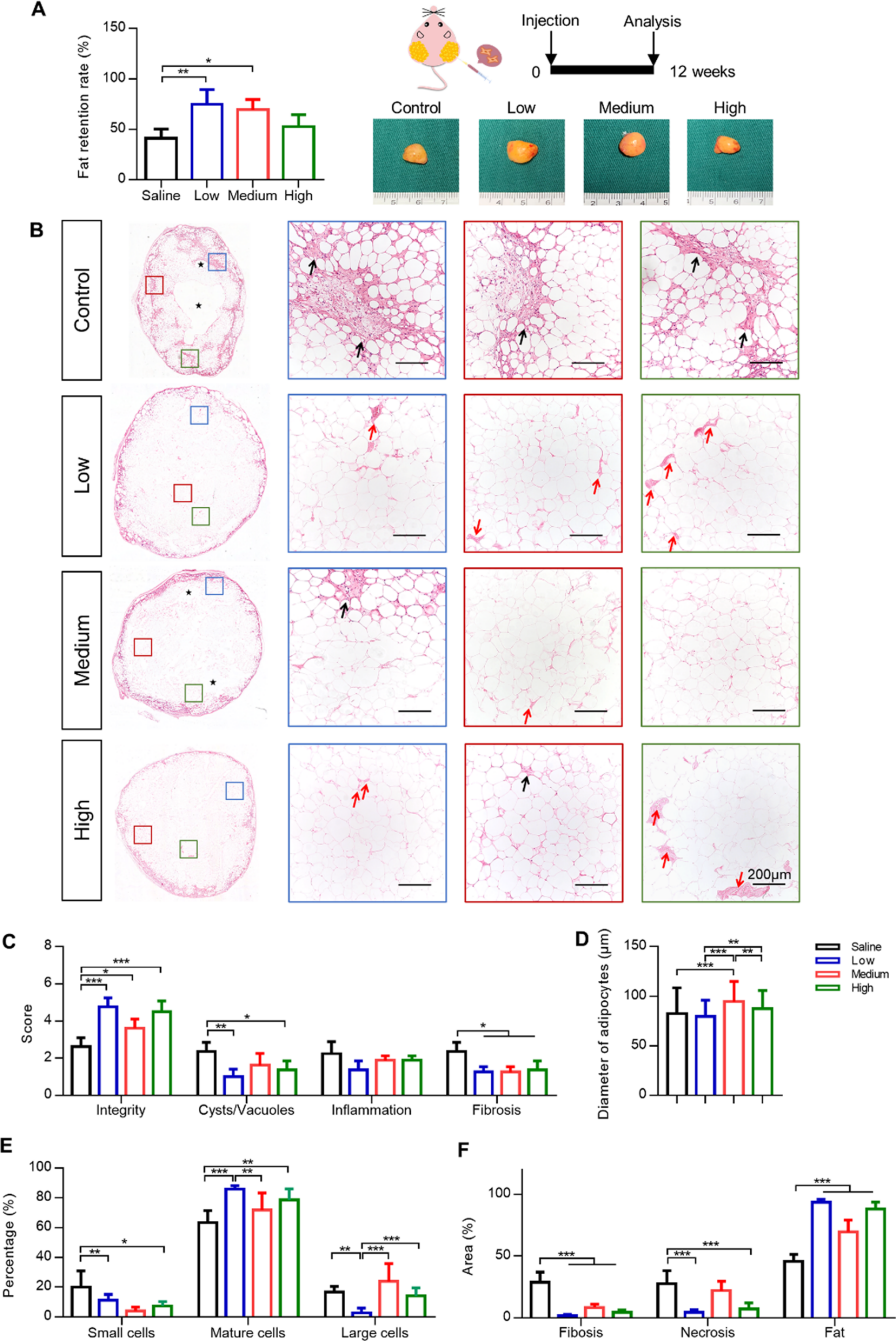
HUCMSCs transplantation promoted the survival of adipose tissue in fat graft without breast scaffold. **A** After 12 weeks of implantation, all the tissue was measured and photographed. The retention rates of grafted fat in the Low hUCMSC group and Medium hUCMSC group were significantly higher than that of the Control group (*P < 0.05, **P < 0.001). **B** HE staining of fat tissue after 12 weeks of implantation (black arrows: fibrosis; stars: cysts or vacuoles; red arrows: blood vessels (scale bars = 200 µm). **C** Histologic evaluation scores demonstrating that hUCMSCs could inhibit necrosis, fibrosis and improve adipose proportion in grafted tissue. **D**, **E** The diameter of adipocytes was measured and classified in different groups, and the low hUCMSC group holds the highest proportion of mature adipocytes among four groups (*P < 0.05, **P < 0.01, ***P < 0.001). **F** The low hUCMSC group holds the lowest cavitary necrosis area and fibrosis degree, but the highest fat tissue occupation among four groups

In order to test the effects of hUCMSCs co-transplantation on breast reconstruction, hUCMSCs were injected into human fat graft without breast scaffold and the implanted tissue were observed after 12 weeks (Fig. 3A). The fat survival rates in the Low group and Medium hUCMSC groups were (75.00 ± 14.47) % and (69.50 ± 9.98) %, respectively, which were significantly higher than the rates reported (41.00 ± 9.31) % in the Control group. However, we found no significant difference between the rates of retained fat (52.50 ± 12.04) % in the High hUCMSC group and Control group.

### Analysis of HE-stained sections of fat grafts

HE staining was carried out to analyze the proportion of tissue components (cyst, fiber, necrosis and fat). We found large cysts and extensive fibrosis in the Control group, whereas all three hUCMSC groups exhibited lower degrees of necrosis, and fibrosis was observed (denoted by black stars and arrows in Fig. 3B). Noticeable vascular structures were found in all three hUCMSC groups (denoted by red arrows in Fig. 3B). Adipose cells were identified microscopically by their typical ring-like morphology, which allowed us to quantify the areas of fibrosis, necrosis, and fat tissue. In all the hUCMSC groups, the scores of adipose integrities were significantly higher and fibrosis was significantly lower than the Control group (Fig. 3C). Since the diameter of normal mature adipocytes ranges from 60 μm to 110 μm [31], the adipocytes could be grouped into three categories, small, mature, and large size adipocytes. Statistical analysis demonstrated that the percentage of mature-sized adipocytes in the Low hUCMSC group was (86.00 ± 2.19) %, the largest ratio in all four groups (Fig. 3E). The percentages of occupied areas of fibrosis, necrosis, and fat in the Low hUCMSC group were (1.73 ± 1.06) %, (4.67 ± 1.85) %, and (93.60 ± 2.40) %, respectively; this demonstrated the least degrees of fibrosis and necrosis, and the largest area of adipose tissue (Fig. 3F). Fat only occupied (45.42 ± 5.96) % area in the Control group which was less than a half of the value in the Low hUCMSC group; the percentages of occupied areas of fibrosis and necrosis were (28.62 ± 8.47) % and (27.63 ± 10.62) %, respectively.

### Immunofluorescence staining of fat grafts

**Fig. 4:**
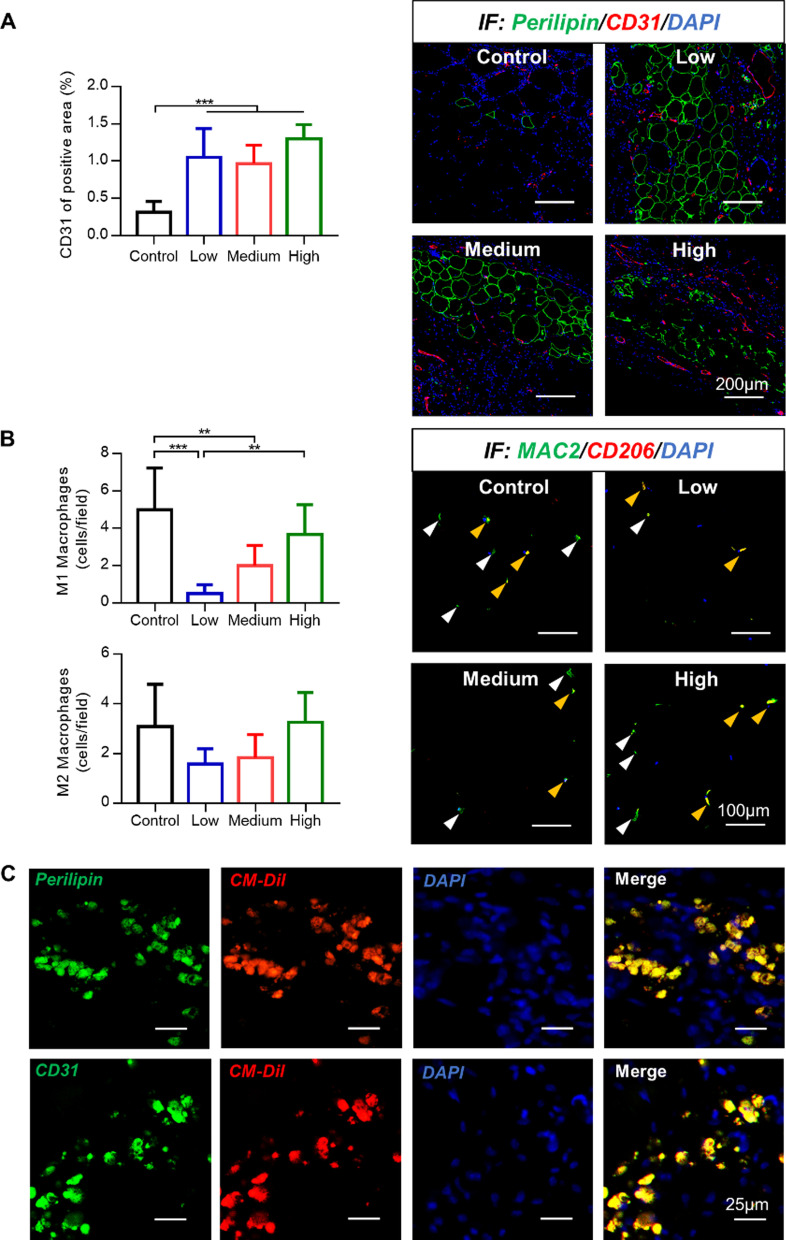
HUCMSCs promoted angiogenesis, attenuated macrophage infiltration, and adipocyte or endothelial differentiation in the adipose tissue. **A** Images of adipocytes (green fluorescence) and blood vessels (red fluorescence). The proportions of CD31^+^ area in each hUCMSC group were significantly higher than the Control group (Scale bars = 200 µm; ***P < 0.001). **B** The distribution of M1 and M2 macrophages (white triangles: M1 macrophages; yellow triangles: M2 macrophages). The numbers of M1 macrophages in the Low hUCMSC group and High hUCMSC group were significantly smaller than the Control group. In terms of M2 macrophages, no significant difference was found between all four groups (scale bars = 100 µm; **P < 0.01, ***P < 0.001). **C** Some transplanted hUCMSCs (CM-Dil-labeled, red fluorescence) were positive expression for Perilipin or CD31 (Scale bars = 25 µm)

Viable adipocytes and endothelial cells were detected through immunofluorescence staining with their specific marker perilipin (green) and CD31 (red). The adipose tissue in the Low hUCMSC group was the most complete, whereas the Perilipin^+^ adipocytes in the Control group were few (Fig. 4A). In all the three hUCMSC groups, proportions of CD31^+^ area vascular endothelial cells were more than three times larger than in the Control group (Control group: (0.31 ± 0.14) %; Low hUCMSC group: (1.05 ± 0.39) %; Medium hUCMSC group: (0.97 ± 0.25) %; High hUCMSC group: (1.30 ± 0.19) %). However, no significant difference was found between the three hUCMSC groups.

Immunofluorescent staining of inflammatory cells demonstrated more MAC2^+^CD206^−^ M1 macrophages (white triangles in Fig. 4B) in the Control group. Statistical analysis revealed that the numbers of M1 macrophages in the Low hUCMSC group and Medium hUCMSC group were significantly smaller than the Control group. However, no significant difference was found between the High hUCMSC group and the Control group (Control group: (5.00 ± 2.23) cells/field; Low hUCMSC group: (0.50 ± 0.47) cells/field; Medium hUCMSC group: (2.00 ± 1.07) cells/field; High hUCMSC group: (3.67 ± 1.59) cells/field). In terms of MAC2^+^CD206^+^ M2 macrophages (depicted by yellow triangles in Fig. 4B), we reported no significant difference between all the four groups.

### hUCMSCs tracing experiment in vivo

CellTracker CM-DiI (red) was used to tract the transplanted hUCMSCs in vivo in the fat grafts after 12 weeks of implantation. Some hUCMSCs with red fluorescence exhibited green fluorescence, indicating that they were positive for Perilipin or CD31 (Fig. 4C). Morphologically, none of these cells showed mature adipocytes or formed vascular structures.

### Breast reconstruction using 3D-printed scaffold and hUCMSC-assisted lipotransfer in vivo

**Fig. 5:**
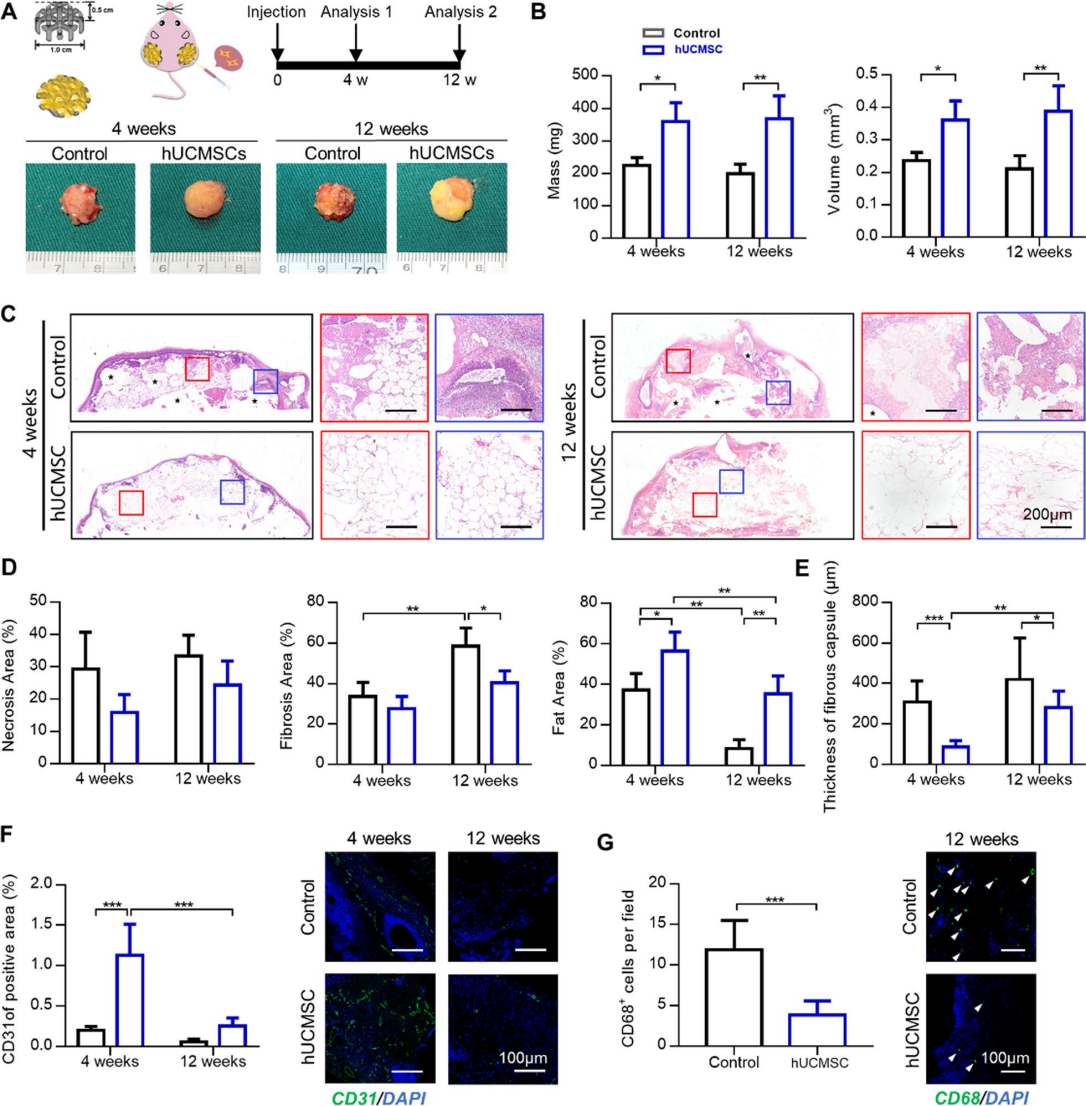
HUCMSCs improved angiogenesis and fat proportion in fat graft with breast scaffold. **A** Scheme of breast tissue engineering construct and the harvested implants. **B** HUCMSCs significantly improved the volumes and mass of the scaffold compounds after 12 weeks of implantation. **C** HE staining of scaffold compounds was shown in (Stars: cysts or vacuoles; Scale bars = 200 µm). **D** HUCMSCs inhibited fibrosis and improved the fat proportion in implants (*P < 0.05, **P < 0.01, ***P < 0.001). **E** HUCMSCs also significantly decreased the thickness of the fibrous capsule of implants (*P < 0.05, **P < 0.01, ***P < 0.001). **F** HUCMSCs significantly increased the area of blood vessels (CD31^+^, green fluorescence) in the engineered breast tissue (scale bars = 100 µm). **G** HUCMSCs significantly decreased the number of macrophages (CD68^+^, green fluorescence, depicted by white triangles) in the implants. (Scale bars = 100 µm)

Finally, hUCMSCs were injected into human fat graft with breast scaffold to evaluate the effect of hUCMSCs co-transplantation on breast reconstruction. After 12 weeks of implantation, we employed a combination of 0.5 × 10^6^ hUCMSCs with 0.5 ml fat and found that the proportion of fibrosis area and cavity necrosis of the fat graft was lower than 7%, whereas the proportion area of adipose tissue was higher than 93% (Fig. 3F). According to the size of the nude mice, the designed breast scaffold was 1 cm in diameter and the maximum volume of fat injected was 0.2 ml. So, in every scaffold, we injected 0.2 × 10^6^ hUCMSCs. After 4 or 12 weeks of the implantation, the scaffold compounds were removed and photographed (Fig. 5A). After 12 weeks of the implantation, the weights of the compounds in the Control group and hUCMSC group were (198.83 ± 29.22) mg and (368.60 ± 70.46) mg, whereas the volumes were (0.21 ± 0.04) ml and (0.38 ± 0.08) ml, respectively. The weight and volume of the compounds in the hUCMSC group were about 1.8 times larger than the Control group (Fig. 5B).

### HE-stained section analysis of tissue engineered breast

Large cysts and severe fibrosis were reported in the Control group, whereas the hUCMSC group exhibited more fat areas and less fibrosis (Fig. 5C). Following quantitative analysis of HE stained images, the area of the fibrosis in the Control group was significantly larger than the hUCMSC group after 12 weeks of implantation (Fig. 5D). However, the area of necrosis between the two groups was not statistically different. In terms of fat area, the percentages in the Control group and hUCMSC group were (37.26 ± 8.11) % and (56.53 ± 9.27) %, respectively, after 4 weeks of implantation. Moreover, the percentages of fat area in the Control group and hUCMSC groups were (8.34 ± 4.33) % and (35.30 ± 8.95) %, respectively, after 12 weeks of implantation. We concluded that hUCMSCs significantly improved the retention rate of fat tissue in the compound. Each implant was characterized by a fibrotic capsule around the compound. The thickness of fibrotic capsule in the Control group was significantly larger than that of the hUCMSC group on both observation time points (4 weeks: (308.67 ± 104.75) μm in Control group, (85.88 ± 32.08) μm in hUCMSC group; 12 weeks: (421.13 ± 204.54) μm in Control group, (280.65 ± 82.69) μm in hUCMSC group) (Fig. 5E).

### Immunofluorescence staining analysis of tissue engineered breast

Immunofluorescent staining of endothelial cells and macrophages was performed to examine the angiogenesis and inflammatory infiltration of the tissue engineering breast. Staining of endothelial cells revealed that the proportion of CD31^+^ area in the hUCMSC group was higher than in the control group after 4 weeks of the implantation. However, the proportion of CD31^+^ area in the hUCMSC group reduced after 12 weeks of implantation, and no statistical difference was found between the two groups (4 weeks: (0.20 ± 0.05) % in Control group, (1.13 ± 0.39) % in hUCMSC group; 12 weeks: (0.06 ± 0.04) % in Control group, (0.25 ± 0.10) % in hUCMSC group) (Fig. 5F). Immunofluorescent staining of CD68 revealed that the number of macrophages in the Control group was about three times larger than in the Control group after 12 weeks of implantation (Control group: (11.83 ± 3.66) cells/field; hUCMSC group: (3.83 ± 1.72) cells/field) (Fig. 5G).

## Discussion

Since the discovery of stem cells, scientists have been devoted their efforts to the replacement of damaged breasts using tissue engineering and regenerative medicine. The adoption of stem cells to replenish the desired breast tissue could eliminate the long-term complications of breast reconstruction, including capsular contracture or donor site morbidity. The benefits of hUCMSCs such as low immunogenicity, non-invasive access, large-scale production, and multilineage differentiation, implicate them as a promising better choice for breast reconstruction. In this study, we found that hUCMSCs could promote vascularization via the integrin β1/ERK1/2/HIF-1α/VEGF-A signal pathway in endothelial cells. hUCMSCs could also directly differentiate into adipocytes and endothelial cells in fat grafts. We adopted the hUCMSCs as seed cells to reconstruct breast with tissue engineering strategy successfully.

In the preclinical studies of various ischemic diseases, hUCMSCs have promoted blood perfusion in ischemic tissues [32, 33]. However, the effects of hUCMSCs on the characteristics of endothelial cells in vitro are unknown. Our results demonstrated that hUCMSC-CM potentially promoted the migration, proliferation, and tubular formation of HUVECs. There was no direct contact between the two cell types in the coculture system, therefore, hUCMSCs may promote vascularization via the paracrine effect.

Furthermore, we attempted to elucidate one of the essential signaling pathways in HUVECs activated by the paracrine secretion of hUCMSCs. Paracrine factors of hUCMSCs have been revealed to promote angiogenesis, such as VEGF-A, bFGF, Ang-1, aFGF, PDGF, etc. [34]. In particular, VEGF-A is the most important of these members as it plays a vital function in both vasculogenesis (de novo vasculature formation) and angiogenesis (vessel formation sprouting from existing vasculature) [27]. VEGF-A ensures the migration of endothelial cells, promotes vessel branching and is indispensable for recruitment, retention, proliferation, migration, and differentiation of endothelial progenitor cells [35]. Besides, VEGF-A expression is intensively influenced by HIF-1α, a transcriptional activator which is unstable under normal condition due to the degradation pathway of the ubiquitin–proteasome pathway [28]. Our results demonstrated that hUCMSC-CM could promote migration, tubule formation and VEGF-A secretion in HUVECs, and which could be suppressed by HIF-1α inhibition.

Integrins, a class of transmembrane proteins consisting of α and β subunits, are greatly involved in cell adhesion, differentiation, growth, migration, and angiogenesis via transmitting extracellular signals across the membrane [30]. Our results showed that the expression of HIF-1α in HUVECs was suppressed followed inhibition of integrin β1. Thus, hUCMSC-CM were suggested to stimulate integrin β1 and then stabilize HIF-1α-mediated VEGF-A expression in HUVECs. ERK1/2 and AKT are common downstream effectors of integrin, and they also participate HIF-1α regulation. Our results demonstrated hUCMSC-CM-induced phosphorylation of ERK1/2, and this could be suppressed by inhibition of integrin β1. Meanwhile, hUCMSC-CM have no affection on AKT activation. Therefore, hUCMSCs potentially promote angiogenesis via paracrine-induced activation of the integrin β1/ ERK1/2/HIF-1α/ VEGF-A signaling pathway in endothelial cells.

Moreover, we evaluated the roles of hUCMSCs injection in fat graft without breast scaffold. The volume retention rates of fat grafts in the Low hUCMSC group and the Medium hUCMSC group significantly improved after 12 weeks of implantation. Of note, the number of hUCMSCs exhibited no potential positive correlation with the volume retention rate of lipotransfer, whereas fat grafts in the Low hUCMSC group owned the highest volume retention rate. Through HE staining and quantitative analysis, we demonstrated that the Low hUCMSC group holds the lowest cavitary necrosis area and fibrosis degree, but the highest fat tissue occupation and the best adipocytes integrity. The proportion of adipocytes within the diameter of mature adipocytes was 86%, and this was the highest among the four groups. Collectively, the adipose tissue in the Low hUCMSC group remained more intact than other groups.

We employed immunofluorescence staining and cell tracing methods to focus on the histological effects and mechanisms of MSC implantation. The immunofluorescence staining of Perilipin confirmed that large areas of complete active adipose tissue of fat grafts in the Low hUCMSC group were preserved, which was consistent with HE staining results mentioned above. Blood vessels were observed through immunofluorescence staining of CD31. Quantitative analysis revealed significantly higher proportions of the red fluorescence (CD31^+^) areas in all three hUCMSC groups compared to that of the Control group. hUCMSCs could, therefore, effectively promote the vascularization and the retention of fat grafts. However, no significant difference in blood vessel areas was found between the three hUCMSC groups. The possible reason might be related to the local inflammation environment following transplantation of different amounts of hUCMSCs. We proceeded with the immunohistochemical staining of macrophages. and revealed that the numbers of M1 macrophages in the Low hUCMSC group and High hUCMSC group were significantly lower than the Control group. Besides, the number of M1 macrophages in the Low hUCMSC group was the least among four groups. The number of M1 macrophages in the High hUCMSC group and the Control group showed no statistical difference but were significantly higher than that in the Low hUCMSC group. For M2 macrophages, we reported no significant statistical difference between all four groups. M1 macrophages express proinflammatory cytokines and are characterized by long-term inflammation, therefore, they could cause tissue damage. Contrarily, M2 macrophages potentially promote tissue remodeling and exert anti-inflammatory and immunoregulatory effects [31]. The degree of fibrosis and calcification of adipose tissue is also related to M2 macrophages [36]. Therefore, a lower amount of hUCMSCs can reduce the number of M1 macrophages to inhibit the inflammatory response, this consequently would reduce the loss of blood vessels and adipocytes.

In addition, we examined the differentiation of hUCMSCs in vivo [37]. Our results showed that numerous hUCMSCs survived in the grafts after 12 weeks of implantation and some positively expressed Perilipin or CD31. The findings confirmed that hUCMSCs could differentiate into adipocytes and vascular endothelial cells in vivo. However, no typical adipocyte morphology or complete vascular ring structure of these hUCMSCs were observed. Therefore, it was concluded that after 12 weeks of implantation, these hUCMSCs might be in the differentiation stage. Whether the differentiated cells exerted the corresponding functions and the stage at which they could change their morphologies into mature cells warrants further exploration.

The present study also explored the effects of hUCMSCs transplantation in breast reconstruction using the combination of lipotransfer and 3D-printed breast scaffolds. TPU materials and 3D printing technology were selected to construct diamond-shaped (N5S4) breast scaffolds. Our previous study demonstrated that the scaffolds with N5S4 architecture that mimic the lattice microstructure of diamond are soft and resilient enough for breast reconstruction [24]. Since the ratio of 0.5 × 10^6^ hUCMSCs to 0.5 ml fat achieves the highest volume retention rate of the graft among all three concentrations and the diameter of the breast scaffold is 1 cm depending on the size of the nude mice, we injected 0.2 ml of fat. The number of hUCMSCs injected was 0.2 × 10^6^ per scaffold. After 4 weeks of implantation, normal breast shape was maintained in the hUCMSC group, while peripheral scaffolds in the Control group were exposed, as such, they lost the normal breast shape. The measure of the weights and volumes of these implants also demonstrated more integrity with the injection of hUCMSCs. Furthermore, HE staining revealed that the proportion of adipose tissue in the scaffolds of the hUCMSC group was significantly higher than that of the Control group, and the degree of fibrosis was significantly reduced. hUCMSCs were suggested to potentially improve the volume retention rate of adipose tissue in the scaffold and inhibit fibrosis. We also noted that adipose tissue in the scaffold reduced from 4 to 12 weeks of transplantation. The immunofluorescence staining of CD31^+^ revealed that the proportion of green fluorescence (CD31^+^) was reduced to 22.12% of the 4-week-value in the hUCMSC group after 12 weeks of implantation. Most of the neovascularization disappeared and at this time point, no significant difference was reported between the hUCMSC group and the Control group. We concluded that hUCMSCs could only promote the rapid neogenesis of blood vessels in the early period of implantation, but stable and mature blood vessels could not be maintained in the long term, thereby reducing the adipose tissue. In addition, surviving fat grafts were associated with exclusively donor-derived vasculature. As the donor-derived microvasculature decreased the graft perfusion at an early stage, large proportions of adipose tissue were lost [38]. Hence, the function of hUCMSCs in promoting rapid vascularization at an early stage is vital to the retention of fat tissue. Also, the number of CD68^+^ macrophages associated with the severe degree of inflammatory response [39] in the tissue engineered breast was significantly reduced after hUCMSCs transplantation. This demonstrated that hUCMSCs could significantly reduce the number of CD68^+^ macrophages in the compound and impeded inflammatory responses, which consequently reduced the damage of adipose tissue. Therefore, to improve the effect of breast reconstruction, hUCMSCs potentially accelerates early vascularization and reduces the inflammatory response.

The clinical therapeutic advantages of stem cells have become increasingly apparent and significant breakthroughs have been made in the management of refractory diseases and tissue engineering [40]. Nevertheless, many stem cells have some limiting factors for their clinical applications, including ethical issues and invasive procedures. HUCMSCs have abundant resources and hold non-invasive extraction procedure [41]. Currently, large-scale production and storage of hUCMSCs are achievable, and hUCMSCs suspension products have been adopted in clinical trials [42, 43]. Importantly, hUCMSCs maintain an earlier embryologic phase, which is much younger and can secrete a wide range of multifunctional factors [34]. Studies have also demonstrated that hUCMSCs are promising in tissue engineering and cell-based therapy. It was once thought that the function of MSCs in the damaged tissue was cell replacement for resident cells, it is now widely confirmed that the more immediate principal mechanism of action of MSCs in vivo is via paracrine [41]. The critical parameters in their ability to modify the function of host cells and tissues are the secretion of multifunctional factors and extracellular vesicles [44]. We have confirmed in the present study that, hUCMSCs can promote vascularization in vitro via paracrine, which potentially activates the vital signal pathway of integrin β1/ERK1/2/HIF-1α/VEGF-A in endothelial cells. HUCMSCs could also differentiate into adipocytes and vascular endothelial cells in fat tissue, and exert immunomodulatory effects by inhibiting the infiltration of M1 macrophages.

## Conclusion

**Fig. 6:**
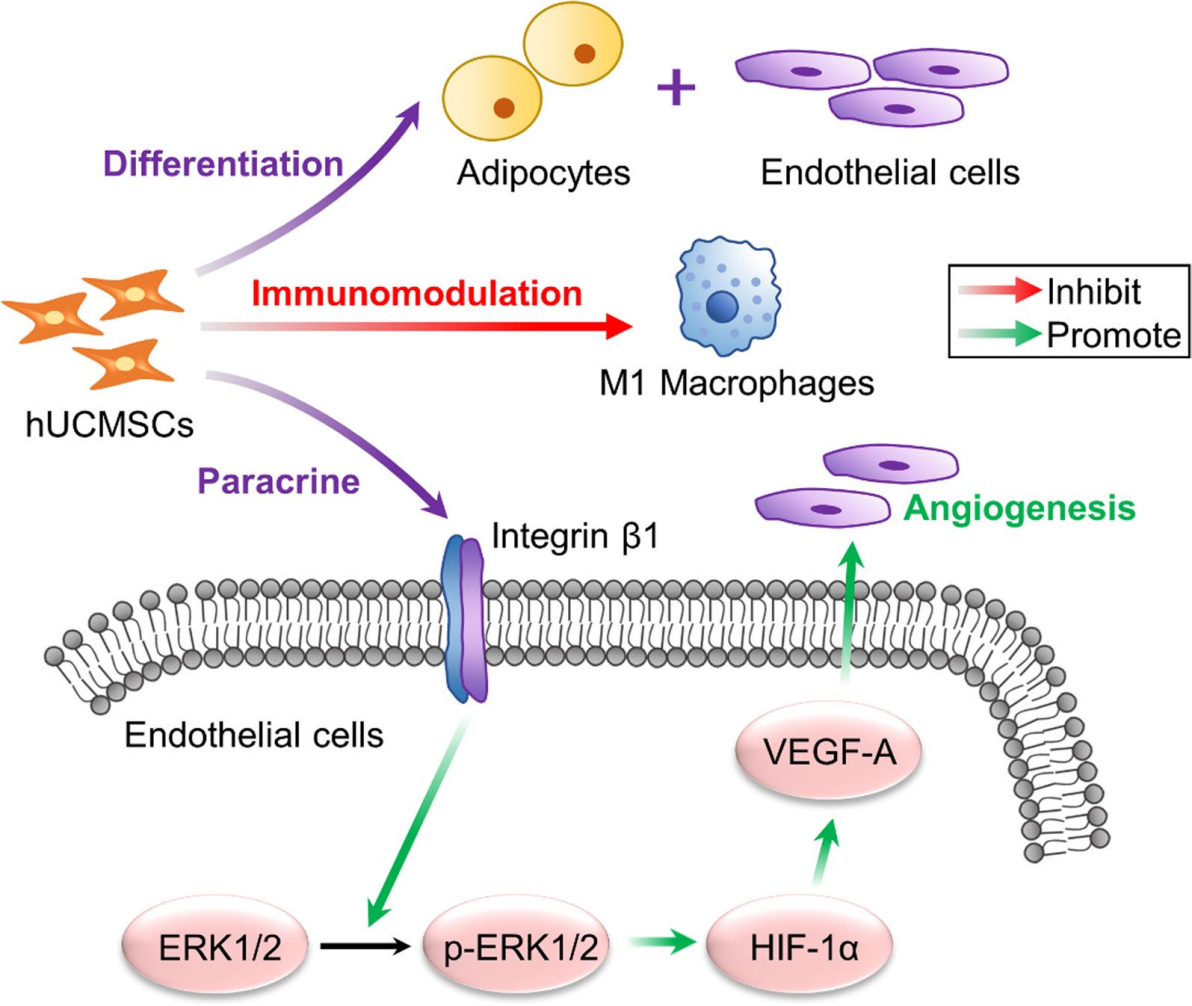
Mechanisms of hUCMSCs injection in breast tissue engineering. HUCMSCs could promote vascularization in vitro via paracrine, which could activate the vital signal pathway of integrin β1/ERK1/2/HIF-1α/VEGF-A in endothelial cells. Moreover, hUCMSCs exerted an immunomodulatory effect by inhibiting M1 macrophages and differentiate into adipocytes and vascular endothelial cells in vivo

In summary, the role and mechanism of hUCMSCs in the tissue engineering breast construction was clarified clearly (Fig. 6). HUCMSCs significantly enhanced proliferation, migration, and angiogenesis of HUVEC through activating the integrin β1/ERK1/2/HIF-1α/VEGF-A signaling pathway in vitro. Also, hUCMSCs were found to improve the volume retention rate of fat tissue by promoting vascularization, inhibiting inflammation, and differentiating into adipocytes and vascular endothelial cells. Additionally, hUCMSCs showed strong capacity to enhance the adipose regeneration and angiogenesis in breast tissue engineering. This study demonstrates the role of allogeneic hUCMSCs in regenerating adipose tissue and provides a new strategy to reconstruct large-volume adipose tissue.

## Supplementary Information

**Additional file 1.** Characterization of hUCMSCs.

## Data Availability

The datasets generated and/or analyzed during the current study are available from the corresponding author on reasonable request.

## Abbreviations

MSC: Mesenchymal stem cells
hUCMSCs: Human umbilical cord mesenchymal stem cells
HUVECs: Human umbilical vein endothelial cells
ADSCs: Adipose-derived stem cells
CM: Conditioned medium
