# Supplementary material for: Human umbilical cord mesenchymal stem cell promotes angiogenesis via integrin β1/ERK1/2/HIF-1α/VEGF-A signaling pathway for off-the-shelf breast tissue engineering
Source: Stem Cell Res Ther. 2022 Mar 7;13:99. doi: 10.1186/s13287-022-02770-x (PMC8900416; doi:10.1186/s13287-022-02770-x)
Supplement: Supplementary file 1 — Additional file 1. Characterization of hUCMSCs. [file 13287_2022_2770_MOESM1_ESM.docx]

**Supplemental Information**


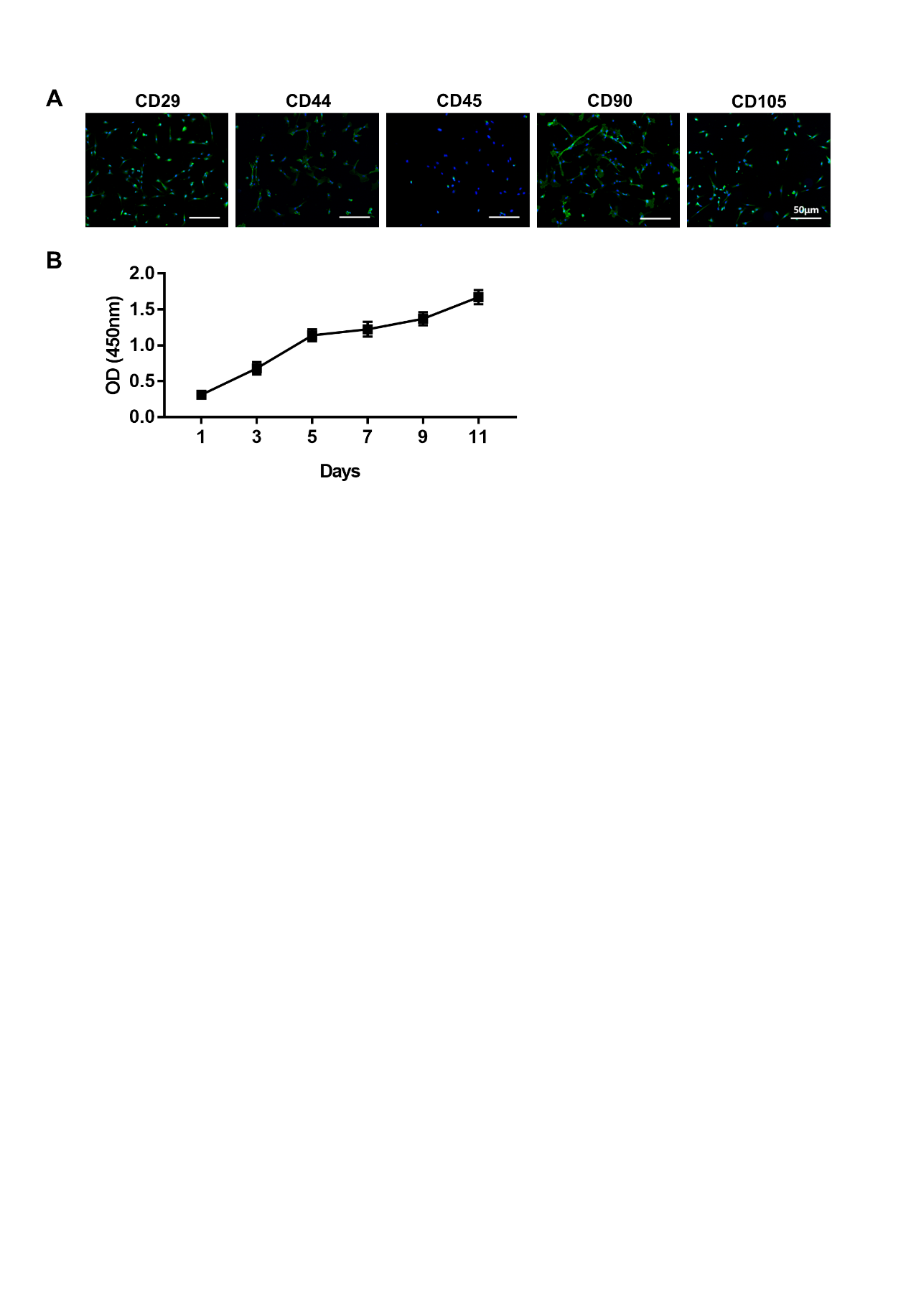


**Fig. S1.** **Characterization of HUMSCs.** (A) HUMSCs were positive for CD29, CD44, CD90, CD105, and negative for CD45. (B) High passage (P8) HUMSCs still showed good proliferative capacity.
